# The Position of the Smaller Institutions

**Published:** 1917-10-06

**Authors:** 


### NURSING AFFAIRS IN IRELAND.

### THE POSITION OF THE SMALLER INSTITUTIONS.

Having regard to the fact, which I reported last week, that county infirmaries in Ireland propose to claim these institutions as nurses' training schools, the subject demands serious consideration. The attitude of the county surgeons who attend these infirmaries is singularly independent, inasmuch as they suggest disregarding what they describe as self-appointed associations, because they apprehend the necessary disfranchisement as nurse-training schools of the hospitals in which they themselves are interested, owing to the inadequacy of the present equipment and want of essential material. Despite this, the county surgeons, however, claim that a hospital containing fifty beds is as potential in respect of training nurses as one containing three times the number. Such a statement as it stands is a palpable absurdity.

### Registration and Recognition.

Perfectly up-to-date statistics are, unfortunately, unobtainable. It might be possible to discover how many patients are treated, and also the ailments, surgical or medical, from which they suffer. But such technical matters as hospital equipment and modernised methods of treatment can only be determined by a public inquiry by professional experts. The question which I wish to bring before your readers is this—if State Registration promises to become a *fait accompli* in the near future, will the Irish county infirmary be able successfully to claim recognition as it stands? In other words, if, for argument's sake, the College of Nursing fixes a minimum limit of beds as a standard for training-schools, is it possible or probable that as the defender and upholder of the rights of the poor, Parliament will lower the standard to a point when up-to-date nurse-training would be rendered impossible by the absence of the necessary material and experience for teachers and taught.

### The Local Government Board's Powers.

There are in Ireland thirty-four county infirmaries, maintained partly by grants from the County Councils, partly by voluntary subscriptions, partly by the proceeds of property held by the male and female governors, and partly by payments from patients. The State makes no contribution. In addition to these thirty-four infirmaries, there are fourteen fever or infectious hospitals, which practically are the fever hospitals for the Unions in which they are situated, and both the county infirmaries and the fever hospitals are the institutions that used to belong to the grand jury system that existed before the Irish Poor Relief Act of 1838. My object in setting out these facts is to show that they all come within the purview of the Irish Local Government Board, on whom will rest the responsibility in Ireland of upholding a nursing standard adequate to protect the rights and claims of Irish men and women when undergoing treatment in these institutions. In any case nurse registration resting on an adequate standard of training could not be made applicable to places where such a standard does not exist.

### A County Hospital Scheme.

It follows that the Local Government Board have a direct interest and responsibility in the training and supply of certificated nurses for the sick poor of Ireland—a fact which is demonstrated by the Report of the Viceregal Commission on Poor-Law Reform in Ireland, a document now out of print. In the Viceregal Report it is recommended that sufficient nurses should be trained and kept in county district hospitals to supply exceptional requirements, so as to make it unnecessary to obtain temporary nurses at considerable expense from a distance. The following is the text of the recommendation:

A county hospital system . . . ought to enable most counties to adopt such a scheme for the training of nurses, partly in the infirmary and partly in the district hospitals and fever hospitals, as would ensure a sufficient supply of efficient nurses throughout each county. Nurses would not be attached to any particular institution, but would be liable to be sent for service to any hospital in the county, and reciprocity as to training facilities might possibly exist between counties, especially in the case of counties of small size or with inconvenient roads of access to the principal hospital of the county. We would suggest that the district hospital authorities, instead of appointing nurses, should nominate probationers. A probationer, on passing for her certificate, would be appointed a county nurse, but possibly with a prior agreement that she should remain in the service of the county for a stated time as nurse. With such a system we would expect a great improvement in county hospitals generally—a levelling up to a high standard.

### The V.-R. Commission's Report Demonstrates the Absence of a Nursing Standard in Irish County Infirmaries and of Material for Adequate Training.

The Commission in question was held in the year 1905, and I am informed on excellent authority that the condition of affairs which then existed approximates very closely to those which now prevail. After careful inquiry the Viceregal Commission referred to pointed out that "it must be admitted that some of the county and union hospitals are very good, some passably good, some middling, some bad, and some very bad." This goes to show that the State, in fixing its requirements, would insist on the maintenance of a high standard. Leaving Dublin and Cork out of account, no county infirmary in Ireland accommodates 100 beds; one 75, twelve between 50 and 70, and several contain less than 30, while one can only boast of 10.

The whole subject is both interesting and important, and demands the early and careful attention of the College of Nursing. Many of the surgeons who attend these county infirmaries are men of eminence in their profession, and, backed by the Local Government Board, will doubtless have a considerable voice in the settling of a standard for the training of nurses in Ireland when State Registration comes before Parliament. IERNE.

